# Improving Nutrition and Physical Activity Policies and Practices in Early Care and Education in Three States, 2014–2016

**DOI:** 10.5888/pcd14.160513

**Published:** 2017-08-31

**Authors:** Teresa M. Smith, Casey Blaser, Cristy Geno-Rasmussen, Julie Shuell, Catherine Plumlee, Tony Gargano, Amy L. Yaroch

**Affiliations:** 1 Gretchen Swanson Center for Nutrition, Omaha, Nebraska; 2 University of Colorado Denver–Anschutz Medical Campus, Denver, Colorado; 3 Nemours Children’s Health System, Washington, DC

## Abstract

The National Early Care and Education Learning Collaboratives (ECELC) project aims to facilitate best practices in nutrition, physical activity, screen time, and breastfeeding support and infant feeding among early care and education (ECE) programs across multiple states. The project uses a train-the-trainer approach with 5, in-person learning-collaborative sessions, technical assistance, and action planning. We describe the longitudinal practice-based evaluation of the project and assess whether ECE programs evaluated (n = 104) sustained changes in policies and practices 1 year after completing the project. The number of best practices increased from pre-assessment to post-assessment (*P* < .01) but did not change significantly from post-assessment to follow-up assessment. ECELC shows promise as an approach to incorporate professional development and training focused on improving best practices for environment-level child nutrition and physical activity, which is one strategy among many that are warranted for obesity prevention in young children.

## Background

In the United States, obesity prevalence among children and adolescents has been about 17% for the past decade (1). Intervention early in life (age 0–5 y) is a key strategy in preventing obesity, because poor diet, physical inactivity, and sedentary behaviors are established well before children enter elementary school (2,3). Nationally, approximately 11 million children aged 5 years or younger spend time in an organized care facility (4), which means early care and education (ECE) programs can be a key setting in which to implement strategies to prevent obesity, targeting children and their families (2). Strategies, including policies and practices related to nutrition (eg, feeding), physical activity, and sedentary behavior (eg, screen time), have potential for environment-level change that can positively influence infants, toddlers, and preschoolers enrolled in these programs (5,6).

In 2007, Nemours Children’s Health System (Nemours) implemented a pilot intervention in Delaware to promote healthy eating and physical activity among children and youth in various settings, including ECE settings. A main component of the initiative included establishing learning collaboratives and train-the-trainer models with ECE programs, which helped these programs identify and implement more healthful policies and practices in the areas of nutrition and physical activity (7). Among the 28 participating ECE programs, 81% significantly increased the number of best practices they met for healthy eating and physical activity (7), suggesting improvement in the environments of young children.

To test whether the pilot model could be successfully scaled up, it was adapted for a multistate implementation in 2012 when the Centers for Disease Control and Prevention (CDC) funded Nemours to launch the National Early Care and Education Learning Collaboratives (ECELC) project. The ECELC aimed to promote healthy environments, policies, and practices in ECE programs as one element of childhood obesity prevention. The original ECELC cohort consisted of 7 sites across 6 states. Data collected demonstrated an increase in the number of best practices (T. M. Smith, PhD, unpublished data, March 2017) followed in these sites: in breastfeeding support and infant feeding (3 additional best practices; *P* < .001), child nutrition (3 to 5 additional best practices, depending on age group served; *P* < .001), infant and child physical activity (2 to 4 additional best practices, depending on age group served; *P* < .001), and screen time (1 to 2 additional best practices, depending on age group served; *P* < .001) as reported by the 511 participating ECE programs immediately after implementation of the ECELC. These findings suggest the ECELC helped foster changes in healthy eating and physical activity policies and practices in ECE programs (T. M. Smith, PhD, unpublished data, March 2017).

The second ECELC cohort, which this article reports on, added 3 new states (225 ECE programs) to the ECELC in May 2014. Lessons learned from the first cohort were used to make minor adjustments to the curriculum and program implementation; however, the program model and main activities remained static. Therefore, a tenable hypothesis is that the second cohort would also demonstrate an increase in the number of best practices followed immediately after participation in the ECELC. However, it was unknown if these changes would be maintained for a period of time after participation in the 10-month ECELC project. Therefore, we sought to examine whether changes made during the ECELC project period were maintained 1 year after the last learning session.

## Evaluation Design

The ECELC is a 5-year cooperative agreement between CDC and Nemours to establish and implement learning collaboratives to support ECE programs in multistate cohorts. The intervention consisted of 5 main strategies: 1) ECE program self-assessment; 2) in-person, peer-learning sessions for ECE teams; 3) action planning and implementation by ECE teams; 4) technical assistance from local trainers; and 5) ECE program reassessment. A longitudinal practice-based evaluation was used to describe the effects of implementing the multistate initiative 1 year after the project ended, with regard to policies and practices related to nutrition, physical activity, screen time, and breastfeeding and infant feeding for programs participating in the ECELC.

This article describes the second cohort of the ECELC, which worked with ECE programs in Kentucky, Virginia, and Los Angeles County, California. ECE-based programs enrolled starting in April 2014 and participated for approximately 10 months. Data on ECE program characteristics (eg, number of children served) were collected electronically when programs enrolled in the ECELC. The primary outcome data were derived from the Nutrition and Physical Activity Self-Assessment for Child Care (NAP SACC) (9), which ECE programs completed after the first ECELC learning session (pre-assessment), approximately 6 to 7 months later after the fourth and penultimate learning session (post-assessment), and then approximately 12 months after the last learning session (follow-up). All ECELC activities ended 3 months after the last learning session, and no further intervention activities were implemented. Evaluation activities were approved by the Nemours Children’s Health System Institutional Review Board.

### Self-assessment of policy and practice data

ECE programs that completed the second cohort of ECELC (ie, were enrolled through the length of the project, completed the pre-assessment, and completed the post-assessment) were eligible for evaluation (n = 189). Eligible ECE programs were recruited via e-mail and subsequently mailed an envelope containing NAP SACC assessments. Programs directors were asked to complete each assessment and return them within 2 months in a self-addressed, stamped, return envelope provided in the packet. Programs received $100 for completing and returning their packets. Programs were sent multiple reminder emails and received telephone calls throughout the 2-month period to encourage response. Reasons for nonresponses included programs having new directors or owners who were unfamiliar with the ECELC, nonworking email addresses, programs having closed since participating in ECE, and main contacts no longer working at programs. Of the 189 eligible programs, 104 (55%) completed and returned the NAP SACC approximately 12 months after the intervention ended.

The NAP SACC consisted of 4 topic areas: breastfeeding and infant feeding (23 items), child nutrition (44 items), infant and child physical activity (22 items), and screen time (12 items) (9). Each item in the 4 areas had 4 response options, ranging from noncompliance (eg, the program offered sugary drinks once per month or more), being close to, but not meeting the best practice (eg, the program offered sugary drinks less than once per month or 1 to 2 times per year) to total compliance with the best practice (eg, the program never offered sugary drinks). Best practices described in NAP SACC include the play environment, feeding practices, staff development, family education, and written policy. For the purpose of this assessment, when the response option representing total compliance was selected, the best practice was considered met (ie, best practice met = 1). All other responses were considered to mean the best practice was not being met (ie, best practice not met = 0).

### Statistical analysis

SAS version 9.4 (SAS Institute, Inc) was used for all statistical analyses. We used paired-sample *t* tests to assess differences between pre-assessment scores and χ^2^ tests for independence to assess characteristics. The sampled data contained complete data on program characteristics, but the number of NAP SACC items per program was dependent on the age groups served by each program.

Means of the total combined NAP SACC, as well as each of the 4 topic areas at pre-assessment, post-assessment, and follow-up, changed from pre-assessment to post-assessment, and change from post-assessment to follow-up was calculated by using the arithmetic mean. Statistical significance of the changes from the post-assessment to 12-month follow-up was assessed by using a repeated measures model, which controlled for program characteristics including age groups served, for-profit status, and participation in the following: Child and Adult Care Food Program (CACFP), Quality Rating and Improvement System, Head Start/Early Head Start, and accreditation programs.

Mean change from pre-assessment to post-assessment was also calculated for all participants of ECELC to date. Statistical significance of the differences in the change of respondent group compared with the entire ECELC was assessed by using *t* tests. Because we found no significant differences across change scores, data for only the participating sample are reported. All statistical significance was set at a 2-sided α level of *P* < .05.

## Changes to Policies and Best Practices

Of the 189 ECE programs that completed both pre-assessment and post-assessment and were therefore eligible to participate in the follow-up assessment, 62% operated as for-profits, 59% participated in CACFP, 46% participated in their state’s Quality Rating and Improvement System, 21% were designated as a Head Start/Early Head Start program, and 15% were accredited by an accrediting agency (eg, National Early Childhood Program Accreditation, National Association for the Education of Young Children, Council on Accreditation) (Table 1). When stratified by programs that responded to the follow-up assessment and those that did not, we found no differences among the groups with the exception of designation as a Head Start/Early Head Start program (14% of respondents vs 30% of nonrespondents; *P* = .007). Additionally, there were no significant differences in pre-assessment NAP SACC scores.

**Table 1:** Characteristics of Early Care and Education (ECE) Programs That Completed the Second Cohort^a^ of the National Early Care and Education Learning Collaboratives Project (n = 189)

Characteristic	Overall	Follow-up Assessment		
		Respondents	Nonrespondents	*P* Value
All programs, N	189	104	85	NA
For profit, %	61.9	59.6	64.7	.47^b^
CACFP, %	58.7	55.8	62.4	.36^b^
QRIS, %	46.0	47.1	44.7	.74^b^
Head Start/Early Head Start, %	20.6	13.5	29.4	.007^b^
Accreditation^c^, %	15.3	18.3	11.8	.22^b^
Combined NAP SACC pre-assessment score^e^, mean	39.1	38.5	39.8	.53^d^
Breastfeeding and infant feeding pre-assessment score^e^, mean	9.9	9.9	10.0	.93^d^
Child nutrition pre-assessment score^e^, mean	23.7	22.9	24.6	.11^d^
Infant and child physical activity pre-assessment score^e^, mean	7.9	7.8	8.0	.79^d^
Screen time pre-assessment score^e^, mean	4.5	4.5	4.5	.90^d^

Abbreviations: CACFP, Child and Adult Care Food Program; NA, not applicable; NAP SACC, Nutrition and Physical Activity Self-Assessment for Child Care; QRIS, Quality Rating and Improvement System.

a ECE programs in Kentucky, Virginia, and Los Angeles County, California, from 2014 through 2015.

b Assessed using χ^2 ^tests for independence.

c ECE program reported whether or not it was accredited (accrediting agency unspecified).

d Assessed using paired *t* tests.

e Each item in this assessment had 4 response options, ranging from noncompliance (being close to, but not meeting the best practice) to total compliance with the best practice. When the response option representing total compliance was selected, the best practice was considered met (ie, best practice met = 1). All other responses were considered to mean the best practice was not being met (ie, best practice not met = 0). Means of the total combined NAP SACC, as well as each of the 4 topic areas at pre-assessment, were calculated by using the arithmetic mean.

Positive changes were seen in the number of best practices met for the combined NAP SACC scores and across all 4 NAP SACC assessments, ranging from an increase of 1.4 best practices (screen time) to 3.6 best practices (child nutrition) (Table 2). All these values were significant at a *P* < .01 level. From post-assessment to follow-up assessment, no significant changes were observed in the number of best practices met for the combined NAP SACC scores or any of the 4 NAP SACC assessments.

**Table 2:** Differences in Changes in NAP SACC Scores From Pre-Assessment to Post-Assessment and Post-Assessment to Follow-Up in Early Care and Education (ECE) Programs That Completed the Second Cohort^a^ of the National Early Care and Education Learning Collaboratives Project (ECELC) (n = 104)

Topic	Mean NAP SACC Scores^b^			Change in Scores^c^		*P* Values	
	Pre	Post	Follow-Up	From Pre to Post	From Post to Follow-Up	From Pre to Post^d^	From Post to Follow-Up^e^
Combined NAP SACC topics	38.5	47.0	48.9	8.6	1.8	<.001	.16
Breastfeeding and infant feeding	9.9	12.6	13.7	2.9	1.0	.001	.28
Child nutrition	22.9	26.5	27.5	3.6	1.0	<.001	.10
Infant and child physical activity	7.8	10.0	9.7	2.2	−0.2	<.001	.56
Screen time	4.5	5.9	6.4	1.4	0.5	<.001	.10

Abbreviation: NAP SACC, Nutrition and Physical Activity Self-Assessment for Child Care.

a ECE programs in Kentucky, Virginia, and Los Angeles County, California, from 2014 through 2015.

b Means of the combined NAP SACC as well as for each of the 4 topic areas at pre-assessment, post-assessment, and follow-up.

c Calculated using the arithmetic mean.

d *P* value for the comparison of the effect of the ECELC intervention between post-assessment and pre-assessment as calculated by a repeated measures model.

e *P* value for the comparison of the effect of the ECELC intervention between follow-up and post-assessment as calculated by a repeated measures model.

## Application of Findings to the Program Model

This sample of ECE programs that participated in the second cohort of the ECELC reported positive and significant changes across all 4 topic areas from pre-assessment to post-assessment, which was also seen in the first cohort (6 states) of the ECELC (8). When the NAP SACC follow-up assessment was repeated approximately 12 months later, there were no significant differences compared with scores reported at post-assessment, generally suggesting that best practices and policies related to nutrition, physical activity, screen time, and breastfeeding support and infant feeding may have been sustained after the intervention ended. Furthermore, it can be posited that since the changes were generally positive but not significant, realistic changes were reported by programs and that changes were not influenced by social desirability bias.

### Public health implications

This evaluation highlights several opportunities for public health interventions in ECE. First, to encourage sustainable change within an ECE program, the NAP SACC was designed to promote policy, practice, and environmental changes (9). Encouraging change at the policy, environmental, and practice levels conceivably helped support change (and potentially sustainability) in the ECE programs that participated in the follow-up evaluation. This concept is modeled in the health impact pyramid, which shows that efforts aimed to address socioeconomic determinants of health via policy may have the greatest impact for change (10). Public health–based policy efforts, coupled with others, such as environment-level interventions that make individuals’ default decisions healthy, are generally more effective than individual-based interventions, because they reach broader segments of society and require less individual effort (10). Of note is that in this intervention, the ECE programs received a great deal of resources and support as part of the ECELC. To translate these findings into practice among non-ECLEC programs, ways to incorporate these learning collaborative and train-the-trainer models into existing infrastructure should be explored (11). We observed in the first cohort of the ECELC a greater increase in number of best practices met among ECE programs that participated in federal programs (eg, CACFP and Head Start) (8), perhaps because of better health and nutrition resources, such as training or guidelines provided in addition to meal reimbursements (8,12–16). Again, considering ways to enact public health–based policy efforts may have the largest impact. Existing government programs may be opportunities to engage ECE programs with training and technical assistance focused on nutrition, physical activity, screen time, and breastfeeding support and infant feeding and facilitate continued support and overall sustainability of efforts (17). The converse may also be helpful, which would be to consider ways to incorporate the learning collaborative and train-the-trainer model employed by the ECELC into existing federal programs to encourage sustained changes made to practices and policies (18).

Nutrition and physical activity initiatives, such as the ECELC, aimed at improving critical environments (eg, ECE) can ultimately contribute to reduced risk for obesity among children under 5 years of age and thus address a major public health problem in the United States. There are no federal nutrition or physical activity standards that are uniformly applied or enforced in ECE programs, and most states lack meaningful regulations related to healthy eating and physical activity (19). Mandating obesity prevention programming in a way that creates additional workload on ECE staff, who are among the lowest-paid workers in the United States and often do not receive health insurance or retirement benefits (20), without augmented compensation and government support could potentially lead to challenges, such as resistance or increased employee turnover (8,21). However, using self-assessment and action planning within the learning collaboratives and train-the-trainer models in ECELC demonstrated success in promoting positive and important changes in ECE, and this model can continue to be adopted or augmented.

### Limitations

Some limitations should be noted. First, data were self-reported via NAP SACC, which is intended to be used in action planning and not as an outcome measure, though data collected via NAP SACC have been reported as outcomes previously (22). Second, some items in NAP SACC were specific to age groups served (ie, infants, toddlers, or preschoolers), whereas the rest were global (ie, applied to all 3 age groups); therefore, a small number of items did not apply to certain programs. Third, even though potential participants were offered compensation for their time, only about half of the population participated in this evaluation. Participants may have been those who were most motivated to integrate change into their ECE programs, so these findings may not be generalizable to the full cohort. Fourth, because NAP SACC pre-assessment occurred after the first learning session and the post-assessment occurred before the last learning session, true pre–post data were not collected. Fifth, given that the same assessment tool was administered at 3 different time points, responses may have been influenced by a learning effect. Lastly, because of time and resource constraints, this evaluation was unable to use a control group, which would have strengthened the design. Despite these limitations, this evaluation shows promise for potential long-term changes to policies and practices in nutrition, physical activity, screen time, and breastfeeding support and infant feeding in ECE settings.

### Conclusions

Integrating obesity prevention programming via a learning collaborative and train-the-trainer model may lead holistically and synergistically toward improved and sustained healthy eating and physical activity policies and practices in ECE programs. Future research should seek to examine and qualitatively extrapolate why some programs do not improve when participating in ECELC or choose to not participate or drop out of the program. Assuring development, implementation, and evaluation of policy and practice-based interventions to help address obesity prevention in specific settings such as ECE can help ensure young children are given equal opportunities for a healthy childhood.
